# Monitoring adherence and patient-reported outcomes during subcutaneous immunotherapy for house dust mites with the AllergyVax app/web platform

**DOI:** 10.1016/j.jacig.2026.100670

**Published:** 2026-02-14

**Authors:** Matheus Fonseca Aarestrup, Fernando Monteiro Aarestrup, Paula Fonseca Aarestrup, Beatriz Julião V. Aarestrup, Edir Paula Cheloni Aarestrup, Akinori Cardozo Nagato, José Otávio Amaral Correa

**Affiliations:** a Allergy and Immunology Department, Suprema Medical School and Health Science, Hospital Maternidade Therezinha de Jesus, Juiz de Fora, Brazil; b Albert Einstein Hospital, São Paulo, Brazil; c Morphology Department, Federal University of Juiz de Fora, Juiz de Fora, Brazil; d Physiology Department, Federal University of Juiz de Fora, Juiz de Fora, Brazil; e Department of Pharmaceutical Sciences, Faculty of Pharmacy, Federal University of Juiz de Fora, Juiz de Fora, Brazil

**Keywords:** Immunotherapy, SCIT, AIT, adherence, outcome, app, web, rhinitis, asthma

## Abstract

**Background:**

Adherence to treatment and outcome monitoring remain major challenges in allergen immunotherapy.

**Objective:**

This study evaluated a digital strategy for monitoring adherence and clinical outcomes of subcutaneous immunotherapy (SCIT) through the AllergyVax mobile and web platform.

**Methods:**

This prospective observational study followed 83 patients receiving SCIT through the AllergyVax digital platform for 12 months. Patient-reported outcomes were assessed with the Rhinitis Control Assessment Test and the Asthma Control Test at baseline and predefined follow-up intervals. Both adherence and clinical outcomes were analyzed longitudinally in the same patient group. Methodologic limitations include absence of a control group and relatively short follow-up.

**Results:**

The assessment of adherence to SCIT revealed that 84.3% of patients adhered to treatment, 7.2% were recovered adherent patients, and 8.4% dropped out after 12 months of treatment. We compared subgroups by analyzing adherence in patients with rhinitis alone versus those with rhinitis and asthma, as well as between male and female patients. None of the comparisons revealed statistically significant differences between the groups.

**Conclusions:**

The digital platform facilitates SCIT management by improving adherence and strengthening patient engagement.

Maintaining adherence to treatment and evaluating clinical outcomes are key challenges in allergen immunotherapy (AIT) for patients with allergic rhinitis and asthma,^1^, ^2^, ^3^, ^4^ with less than half completing the treatment.^1^ A systematic review reported that real-world adherence rates for subcutaneous immunotherapy (SCIT) are low.^5^ Mobile technologies offer a promising approach to enhance the doctor–patient relationship and improve adherence to AIT.^3^^,^^4^ The use of patient-reported outcome (PRO) measures to monitor clinical results fosters patient engagement and satisfaction by providing a clearer sense of treatment efficacy.^6^, ^7^, ^8^, ^9^ A recent real-world study using the AllergyVax app/web platform demonstrated that mobile technology could improve adherence to sublingual immunotherapy when integrated into a patient-centered care (PCC) strategy for managing AIT.^9^

The AllergyVax app/web platform (AllergyVax USA, Houston, Tex; and AllergyVax Brazil, Juiz de Fora, Minas Gerais, Brazil) was developed to support the management of AIT. It holds trademark registration in both the United States and Brazil and is available in the Apple Store and Google Play Store in Brazil, Europe, and the United States. The platform has been validated by Apple and Google, and it complies with security, privacy, and device protection standards. It ensures the privacy of user data and adheres to both local and international laws regarding intellectual property and metadata. The app offers a free version for patients and a professional subscription for physicians. It supports English, Portuguese, and Spanish. The AllergyVax platform automates general operations in a health care setting.

The primary objective of this study was to implement a digital strategy to monitor adherence and outcomes of SCIT when the AllergyVax app was used. The secondary objectives were to evaluate SCIT adherence and PROs, specifically scores for the Rhinitis Control Assessment Test (RCAT) and Asthma Control Test (ACT).

## Methods

We conducted a prospective observational cohort study involving 83 patients from the Allergy and Clinical Immunology Service in Juiz de Fora, Minas Gerais, Brazil.^10^^,^^11^ The research ethics committee of Suprema Medical School and Health Science, Juiz de Fora, approved the study (registration 1481788). Eligible patients were at least 12 years old, had a confirmed diagnosis of mild to moderate rhinitis and/or asthma established by an allergy and immunology specialist, and had initiated SCIT (IPI/ASAC, São Paulo, Brazil) specifically for *Dermatophagoides pteronyssinus* and/or *Blomia tropicalis* on the basis of skin prick test results. Diagnosis and treatment followed the Allergic Rhinitis and Its Impact on Asthma and the Global Initiative for Asthma guidelines, and all patients or their guardians provided informed consent before initiating SCIT.

The study used the Portuguese version of the AllergyVax professional app for physicians. Patients and/or guardians accessed and accepted the “Terms and Conditions/Privacy” section of the AllergyVax app, which outlined data use, storage, and confidentiality. A nurse provided in-person training and demonstrated the app’s features, ensuring participants understood the procedures before enrollment. Data recorded in the platform were analyzed to measure SCIT adherence 12 months after initiation. Patients underwent a 5-week buildup: Each week they received 3 sequential same-day SCIT injections, reaching the maintenance dose in week 5. During maintenance, injections were administered every 15 days and then monthly from month 3 onward.

All SCIT injections were administered at the clinic under the supervision of a physician and clinical staff. At each visit, patients completed PRO measures, and the results were immediately available and automatically sent via the platform to the email of both the physician and the patient or legal guardian. SCIT was administered only after verification of the PRO results. Patients whose disease was not clinically controlled were examined by the physician beforehand to assess any potential contraindications and to determine the need for medication or adjustments to the immunotherapy schedule.

We monitored SCIT adherence and outcomes through the platform. Adherence was defined as completing 12 consecutive months of treatment with a maximum delay of up to 2 weeks between applications. Patients who had a treatment gap of 2 to 4 weeks but restarted and maintained SCIT until month 12 were classified as adherence recovered. PROs were assessed using the RCAT and ACT at baseline and at regular follow-up intervals. In this observational cohort, outcomes and treatment adherence were tracked over time in the same patients, without a formal control group.

Table I summarizes the tasks automated by the AllergyVax app/web platform. The system automatically sends alerts for missed or delayed SCIT injections via email and text messages to the physicians, patients, and clinical staff. The purpose of this automation is to facilitate patient follow-up, prevent treatment abandonment, and avoid delays that may compromise therapeutic outcomes. When SCIT injections are delayed, physician evaluate patients to determine whether dosing adjustments are needed before treatment can continue. Clinical staff also contact patients who fall behind schedule and encourage them to reschedule their SCIT appointments. Medical records document the reasons for any modifications, and dose adjustments follow national guidelines.

**Table I:** Summary of automated functions provided by AllergyVax app/web platform to support SCIT adherence and outcome monitoring

Function	Description
Reminder alerts	• Automatic email and text message reminders sent 1 week and 1 day before each SCIT appointment. • Email alerts and text message to doctor and patient when SCIT sessions are missed or delayed. • Emails to patients reminding them to schedule missed SCIT appointments. • Weekly summary emails to doctors and staff listing patients with delayed SCIT.
PRO monitoring	• RCAT and ACT questionnaires, with results automatically emailed to both doctor and patient.
Patient support	• Provides information and guidance about AIT. • Includes web links to major allergy and clinical immunology associations.

Table II presents the profile of the study population, categorized by diagnosis, sex, and age. The study included patients diagnosed with persistent allergic rhinitis (mild, moderate, and severe) or persistent allergic asthma (mild and moderate), classified according to the Allergic Rhinitis and Its Impact on Asthma and the Global Initiative for Asthma guidelines. Pharmacologic treatment was used only when necessary, in strict accordance with the stepwise recommendations of the guidelines. Medications were prescribed for the minimum period required to obtain clinical control, after which patients continued exclusively with SCIT. This approach ensured that SCIT remained the primary long-term intervention while maintaining guideline-based management of symptoms during exacerbations.

**Table II:** Frequency distribution and relative percentage of patients with rhinitis or rhinitis and asthma treated with SCIT and monitored for 12 months with AllergyVax app

Characteristic	Rhinitis		Asthma and rhinitis		*P* value	DM†	95% confidence interval	η^2^	Total
	Value	Present	Value	Present					
Male	29.00 (69.05)		13.00 (30.95)						42.00 (100.00)
Age (years) (normality test)	25.10 ± 2.58	Yes	27.54 ± 2.78	Yes	.57	2.44 ± 4.29	−6.24 to 11.11	.01	
Regular visits (normality test)	9.21 ± 0.57	No	8.69 ± 0.64	Yes	.59	−.51 ± .96	−2.45 to 1.42	.01	
RCAT or ACT score (normality test)	23.26 ± 0.56	Yes	22.16 ± 0.73	Yes	.27	−1.10 ± .97	−3.06 to 0.87	.03	
Adherence	22.00 (75.86)		11.00 (84.62)						33.00 (78.57)
Recovered	2.00 (6.90)		2.00 (15.38)						4.00 (9.52)
Adherence + recovered	24.00 (82.76)		13.00 (100.00)						37.00 (88.10)
Dropout	5.00 (17.24)								5.00 (11.90)
Female	29.00 (70.73)		12.00 (29.27)						41.00 (100.00)
Age (years) (normality test)	33.52 ± 2.89∗	Yes	34.00 ± 3.46	Yes	.92	.48 ± 5.03	−9.69 to 10.66	.00	
Regular visits (normality test)	8.35 ± 0.49	Yes	10.17 ± 0.49	No	.03	1.82 ± .83	0.15 to 3.49	.11	
RCAT or ACT score (normality test)	21.96 ± 0.67	Yes	22.53 ± 0.84	Yes	.64	.56 ± 1.18	−1.82 to 2.95	.06	
Adherence	25.00 (86.21)		12.00 (100.00)						37.00 (90.24)
Recovered	2.00 (6.90)		—						2.00 (4.88)
Adherence + recovered	27.00 (93.10)		12.00 (100.00)						39.00 (95.12)
Dropout	2.00 (6.90)								2.00 (4.88)
Male and female	58.00 (69.88)		25.00 (30.12)						83.00 (100.00)
Age (years) (normality test)	29.31 ± 2.00	Yes	30.64 ± 2.25	Yes	.70	1.33 ± 3.39	−5.42 to 8.08	0	
Regular visits (normality test)	8.78 ± 0.38	No	9.40 ± 0.43	Yes	.33	.62 ± .64	−0.65 to 1.90	.01	
RCAT or ACT score (normality test)	22.61 ± 0.44	Yes	22.34 ± 0.54	Yes	.72	−.27 ± .76	−1.79 to 1.25	0	
Adherence	47.00 (81.03)	Yes	23.00 (92.00)	Yes					70.00 (84.34)
Recovered	4.00 (6.90)		2.00 (8.00)						6.00 (7.23)
Adherence + recovered	51.00 (87.93)		25.00 (100.00)						76.00 (91.57)
Dropout	7.00 (12.07)								7.00 (8.43)

Data are presented as nos. (%) or mean ± SE unless otherwise indicated. Patients comprised 83 subjects who received guidelines and information through traditional methods (without AllergyVax app). RCAT was used to monitor patients with rhinitis, and ACT was used to monitor patients with asthma. RCAT range was 6-30; ACT range was 5-25. Cutoff values for controlled symptoms were ≥22 (RCAT) and ≥20 (ACT). Normality of parametric data was tested by D’Agostino and Pearson test. Statistically significantly different (*P* < .05) at 95% confidence interval by unpaired *t* test. Difference between parametric data was tested by unpaired *t* test.

† Difference between means (asthma and rhinitis − rhinitis) ± SEM.

∗ *P* = .034 compared to age/male (η^2^ = .07761). Other comparisons taken 2 by 2 showed no significant difference. There was no difference between other groups.

All information was provided exclusively by patients or their guardians, and data collection occurred automatically without our interference. Statistical analyses were performed without identifying the patient groups, ensuring confidentiality and reliability of the information and results.

The RCAT was used to monitor patients with rhinitis and the ACT was used to monitor patients with asthma. The qualitative patient data were expressed as frequency distribution with relative percentage. The RCAT and ACT ranges were 6-30 and 5-25, respectively. The cutoff values for controlled symptoms were ≥22 for the RCAT and ≥20 for the ACT. The normality of the parametric data was tested by the D’Agostino and Pearson test. The parametric data were expressed as arithmetic mean ± SEM. The probability of differences between groups was considered when *P* < .05 for a 95% confidence interval, as determined by an unpaired *t* test. The difference between the intragroup means for asthma or rhinitis and asthma was accompanied by ±SEM, and the *R*^2^ value was tested by the chi-square test through contingency analysis. Statistics and graphs were created by GraphPad Prism v8.4 for Windows (GraphPad Software, La Jolla, Calif). All data were preserved, and no missing data were identified.

## Results

**Fig 1:**
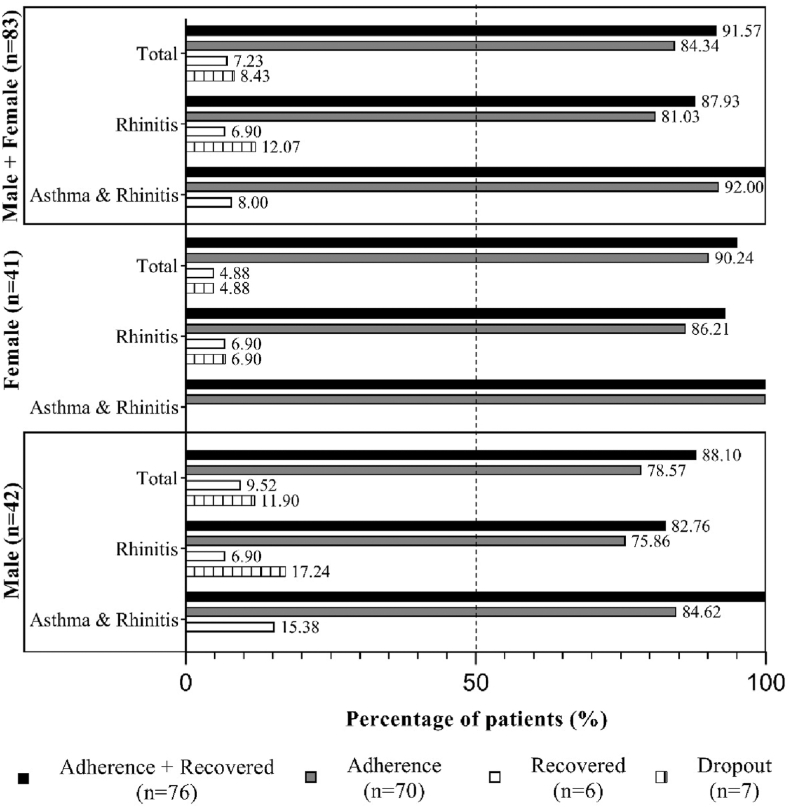
Frequency and percentage distribution of patients (n = 83) who adhered to (n = 70), recovered from (n = 6), or dropped out of SCIT (n = 7) after 12 months of follow-up using AllergyVax platform, categorized by sex (male, n = 42; female, n = 41).

**Fig 2:**
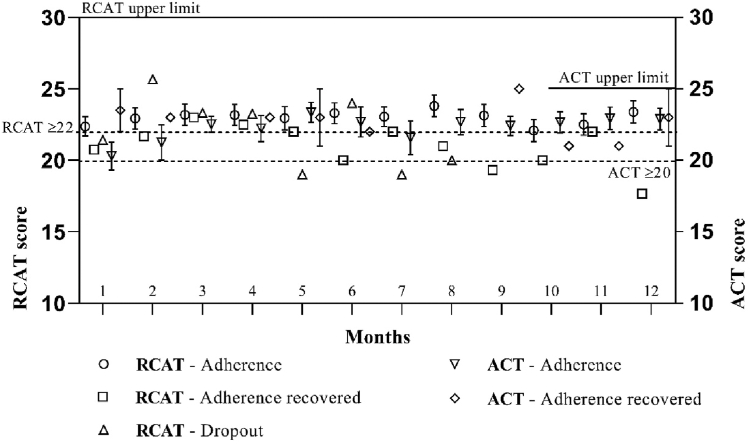
RCAT and ACT scores of patients (n = 83) who received standard guidance and were monitored via AllergyVax platform over 12 months. Score ranges were 6-30 for RCAT and 5-25 for ACT; cutoff values for controlled symptoms were ≥22 (RCAT) and ≥20 (ACT).

Fig 1 and Table III show high adherence to treatment among patients with rhinitis or with rhinitis plus asthma regardless of sex. Fig 2 illustrates the evolution of PRO scores (RCAT and ACT) across adherence subgroups: adherence, adherence recovered, and dropout.

**Table III:** Frequency distribution and relative percentage of patients who were treated with SCIT and were followed up for 12 months with AllergyVax app

Characteristic	Rhinitis	Asthma and rhinitis	Total
Male	29.00 (69.05)	13.00 (30.95)	42.00 (100.00)
Mild	5.00 (17.24)	2.00 (15.38)	7.00 (16.67)
Moderate	7.00 (24.14)	11.00 (84.62)	18.00 (42.86)
Severe	17.00 (58.62)	0	17.00 (40.48)
Female	29.00 (70.73)	12.00 (29.27)	41.00 (100.00)
Mild	0	4.00 (33.33)	4.00 (9.76)
Moderate	14.00 (48.28)	8.00 (66.67)	22.00 (53.66)
Severe	15.00 (51.72)	0	15.00 (36.59)
Male and female	58.00 (69.88)	25.00 (30.12)	83.00 (100.00)
Mild	5.00 (8.62)	6.00 (24.00)	11.00 (13.25)
Moderate	21.00 (36.21)	19.00 (76.00)	40.00 (48.19)
Severe	32.00 (55.17)	0	32.00 (38.55)

Data are presented as nos. (%).

Symptom control was consistently maintained throughout the 12 months of treatment, as demonstrated by RCAT and ACT results (Fig 2). All patients with rhinitis plus asthma who experienced treatment delays of 2 to 4 weeks successfully recovered adherence. However, most of adherence-recovered patients with rhinitis plus asthma presented partially controlled or uncontrolled asthma (ACT ≤ 20) at some point during follow-up.

Local and systemic reactions were assessed by clinicians. Mild reactions with pruritus and hyperemia were occasionally observed at the SCIT application site. Only one patient had an episode of extensive local reaction, which required antihistamines and oral corticosteroids. No systemic reactions were observed during the study period.

Table III shows the percentage of adherence among all patients. The assessment of adherence to SCIT revealed that 84.3% of patients adhered to treatment, 7.2% were recovered adherent patients, and 8.4% dropped out after 12 months of treatment. We compared subgroups by analyzing adherence in patients with rhinitis alone versus those with rhinitis and asthma, as well as between male and female patients. We applied chi-square and *t* tests, setting statistical significance level at *P* < .05. None of the comparisons revealed statistically significant differences between the groups. The mean scores of the RCAT and ACT analyzed by sex showed that sex had no effect on the mean scores observed (*P* = .9472, 1-way analysis of variance). The mean ± standard error of the RCAT among women was 22.76 ± 2.90 and among men was 22.47 ± 3.61, while the mean ACT scores among women were 22.02 ± 2.97 and among men were 22.51 ± 2.44. These results indicate that the level of clinical control of patients with rhinitis and rhinitis and asthma was similar between men and women, showing consistent patterns in the scores in both conditions.

## Discussion

The European Academy of Allergy and Clinical Immunology recommends technology-driven PCC as a way to enhance clinical practice, especially by improving adherence and evaluating treatment outcomes.^5^ Mobile health technologies have become increasingly relevant in managing allergic diseases, offering effective tools to strengthen adherence and monitor clinical results.^12^, ^13^, ^14^

The AllergyMonitor app has shown that electronic diaries can effectively track symptoms and adherence to AIT in real-world settings, helping both patients and clinicians optimize care. Likewise, the MASK-air app has been validated in multiple countries as a reliable tool to assess rhinitis control, compare treated and untreated patients, and develop combined symptom–medication scores as potential end points in AIT trials.^12^^,^^14^ Building on these experiences, a recent real-world study introduced AllergyVax, a dedicated app/web platform developed to monitor adherence and evaluate PROs during AIT.^9^

The high adherence to SCIT observed in this study likely reflects the combination of strategies implemented through the AllergyVax platform, which actively engaged patients, physicians, and clinical staff while keeping the patient at the center of care. The platform carries out several automated actions to support treatment management, including email reminders sent 1 week and 1 day before each SCIT appointment, automatic alerts to both patients and physicians when sessions are missed or delayed, and the administration of RCAT and ACT questionnaires with immediate electronic delivery of results.

In the present study, adherence was rigorously defined to include the concept of *recovered adherence,* which considers patients who experience temporary interruptions but subsequently resume and maintain SCIT through the twelfth month. This operational approach mirrors real-word clinical practice; brief delays or interruptions should not automatically disqualify patients from being considered adherent when therapy is ultimately continued. The professional version of the AllergyVax platform provides physicians with real-time information on each patient’s adherence status and PRO results. Continuous monitoring of AIT adherence and clinical outcomes enables timely and targeted interventions when progress is suboptimal, allowing treatment plans to be adjusted to optimize symptom control during AIT. Our findings suggest that patients’ own perceptions of symptom control, as captured through PRO questionnaires, may play a key role in sustaining adherence and facilitating the recovery of those who experienced temporary interruptions in therapy. Although this study did not investigate specific reasons for treatment discontinuation, previous literature identifies two major contributors: lack of perceived efficacy and financial constraints.^1^^,^^5^^,^^15^ In our cohort, the favorable outcomes observed likely contributed to the low dropout rate, as positive responses tend to reinforce patient engagement.

The professional subscription version of AllergyVax offers a set of exclusive, high-impact features that go far beyond symptom tracking, uniquely supporting AIT (SCIT and sublingual immunotherapy). In addition to monitoring treatment outcomes and adherence with precise longitudinal tracking, the platform performs symptom follow-up using validated PRO measures, including the ACT and the RCAT, which are scientifically validated instruments widely used in routine clinical practice and research. This level of validated clinical monitoring is not implemented in MASK-air or AllergyMonitor, whose symptom tracking relies mainly on daily logs, visual analog scale scores, and medication diaries rather than standardized PROs.^3^^,^^12^^,^^14^ Moreover, AllergyVax provides a comprehensive suite of automated communication tools that send reminders, safety instructions, and personalized messages directly to patients, greatly improving adherence and continuity of care.^9^ Ultimately, AllergyVax aims to support both the clinical and administrative management of AIT, with a strong focus on improving adherence and implementing a comprehensive PCC strategy.

This study has some limitations. It was conducted in a single cohort without randomization, and although the follow-up period was sufficient to assess adherence trends, it may not capture the long-term outcomes of AIT. The Centre for Evidence-Based Medicine emphasizes that randomized controlled trials are not always necessary or feasible, and observational studies can be more appropriate when randomization is not possible.^10^^,^^11^ In this context, the absence of a formal control group represents a minor limitation compared with randomized controlled trials. A key strength of this study is the longitudinal evaluation of treatment adherence and PROs over time.

Although the patient sample is small, the results observed showed consistency and reproducibility throughout the entire evaluation period. The results observed are the consequence of a set of strategies facilitated by the AllergyVax app, which made the patient an active participant in the AIT treatment process. Overall, our findings suggest that this innovative digital tool can support the clinical management of SCIT by promoting adherence and enhancing patient engagement, thereby potentially improving therapeutic outcomes. Future studies with larger cohorts, longer follow-up, and comparative designs are needed to validate these results and assess the scalability of the digital strategy across diverse clinical contexts.

The consistent improvement in PRO scores during follow-up reinforces the clinical benefits of sustained SCIT and highlights the potential of digital health solutions to foster patient engagement. The increase in treatment adherence was not solely a result of using the AllergyVax app. It is likely that the increase in adherence was the result of a care strategy in which mobile technology facilitated the entire process, including communication, education, and patient motivation, as well as assisting in various clinical and administrative activities related to AIT. By actively involving patients in tracking their own progress, platforms such as AllergyVax may help bridge the gap between prescribed treatment and long-term adherence. These findings align with recent literature emphasizing the role of digital platforms in chronic disease management, particularly in conditions that require prolonged interventions.

**Key messages**

• This study showed the clinical relevance of the AllergyVax app/web platform as a digital tool that integrates automation and PRO measures to improve adherence and optimize SCIT management.
• By combining automated reminders, real-time PRO monitoring, and data sharing between patients and clinicians, the platform promotes PCC, strengthens engagement, and allows timely treatment adjustments.
• These features streamline workflow, enhance communication, and support sustained adherence, ultimately contributing to more personalized care in routine practice.

## Disclosure statement

Declaration of generative AI and AI-assisted technologies in the report’s preparation process: During the preparation of this work, the authors used ChatGPT in order to correct formatting and language. After using this tool/service, the authors reviewed and edited the content as needed and take full responsibility for the content of the published report.

Disclosure of potential conflict of interest: F. Monteiro Aarestrup is a developer of the AllergyVax app/web platform; no personal fees or grants were received in connection with the development of the study. The rest of the authors declare that they have no relevant conflicts of interest.
